# Prevalence of Epstein–Barr virus (EBV) in Iranian Breast Carcinoma Patients

**DOI:** 10.31557/APJCP.2020.21.1.133

**Published:** 2020

**Authors:** Morvarid Golrokh Mofrad, Behrang Kazeminezhad, Ebrahim Faghihloo

**Affiliations:** 1 *Human Viral Vaccine Department, Razi Vaccine and Serum Research Institute, Agricultural Research, Education and Extension Organization (AREEO), Karaj,*; 2 *Department of Pathology, *; 3 *Department of Microbiology, School of Medicine, Shahid Beheshti University of Medical Sciences, Tehran, Iran. *

**Keywords:** Epstein, barr virus, breast cancer, PCR

## Abstract

**Introduction:**

Breast cancer (BC) is the most common malignancy affecting females worldwide. Various risk factors play a role in the developing of BC. Infectious agents like viruses have been proposed for this cancer and Epstein-Barr virus (EBV) is a widely researched candidate virus. This study detects the presence of EBV-DNA in breast cancer patients.

**Methods:**

The study was conducted on 59 formalin-fixed paraffin-embedded (FFPE) tissue blocks samples of women with breast carcinoma and 11 non-neoplastic breast controls. The DNA was extracted for all the samples. Then detection of EBNA1 EBV was done by polymerase chain reaction (PCR).

**Results:**

EBV was detected in 6.7% (4/59) of patients while all breast control samples were negative. All patients with positive EBV-DNA were high tumor grades (II, and III). Also, they had a low level of educations.

**Conclusions:**

According to our findings, it can be suggested that EBV may have a potential role in breast cancer development. However, this study provides substantial but not conclusive evidence for the involvement of viruses in BC disease development. Therefore, future investigations are needed to elucidate the exact role of EBV in breast cancer.

## Introduction

Breast Cancer (BC) is one of the most prevalent cancers among females and also it’s the primary cause of high morbidity and mortality due to malignancies in the world. There is an enormous difference in breast cancer survival rates worldwide, with an evaluated 5-year survival of 80% in developed countries to below 40% for developing countries (DCs) (Coleman et al., 2008).In the US, this rate is about 90%. However the incidence of breast cancer is lower in DCs, but the ratio of the mortality is higher(Parkin et al., 2005; Allemani et al., 2015). Roughly 60% of deaths due to breast cancer occur in DCs, whereas in developed countries, more than 200 cases per 100,000 women are diagnosed per year (van Netten et al., 1987; Tsutsui et al., 2002; Talvensaari-Mattila et al., 2003; Torre et al., 2015). Unfortunately, breast cancer is diagnosed in the late stages in DCs because in these countries early detection, diagnosis, and treatment cannot be adequately promoted (Anderson et al., 2006). Breast cancer can be metastatic cancer and can frequently move to other organs such as the bone, liver, lungs, and brain, which mostly responsible for its incurable potential. Early diagnosis can lead to a worthy prognosis and a high survival rate (DeSantis et al., 2016).

BC is a multifactorial disease and various risk factors for this disease have been identified, like age, geographical variation, age at menarche and menopause, age at first pregnancy, family history, previous benign breast disease, radiation, lifestyle (includes : Diet, Weight, Alcohol intake, Smoking) and oral contraceptive (Kamińska, Ciszewski et al., 2015). Some viruses such as human cancer such as human papillomaviruses and Epstein-Barr virus have been implicated in the pathogenesis of breast cancer (Akhter et al., 2014).

Epstein - Barr virus (EBV) is one of the most common human viruses and infects about 95% of the world’s population (Khan and Hashim, 2014). It’s a ubiquitous gamma herpesvirus causes infectious mononucleosis and also it is related with the development of different Malignancies like Burkitt’s lymphoma, Hodgkin’s disease, B-cell lymphoma in immunocompromised individuals, nasopharyngeal carcinoma (NPC) and T-cell lymphoma, gastric carcinoma, and thymus and lung malignancies (Niedobitek, 2000; Takeuchi et al., 2004; Khan and Hashim, 2014; Oh and Weiderpass 2014; Gru et al., 2015). 

EBV can entry to epithelial cells with the help of ligands that exists on the surface of B-cells, called CD21 or CR2 or EBVR (Borza and Hutt-Fletcher, 2002). EBV infection is mostly latent in target cells. EBV latency is characterized by a limited expression of viral proteins (latency III): i.e., six nuclear proteins EBNA-1, EBNA-2, EBNA-3A-C, EBNA-LP, and three latent membrane proteins (LMP-1, LMP-2A, and LMP-2B) (Kang and Kieff, 2015). 

Different laboratories have reported the detection of EBV in a subset of breast tumors (Labrecque et al., 1995; Luqmani and Shousha, 1995; Bonnet et al., 1999). Although, negative results have also been reported (Dadmanesh et al., 2001; Deshpande et al., 2002; El-Naby et al., 2017). EBV infection inclines breast epithelial cells to malignant transformation through activation of HER2/HER3 signaling cascades. HER2 and HER3 are two of the cellular oncogenes known to be involved in human breast cancer development (Hu et al., 2016).

A large number of epidemiological studies have shown the association between EBV infection and breast carcinoma like a meta-analysis study of Huo Q et al. So the result was remarkable, they found 29.32% of the patients with BC were infected with the Epstein-Barr virus. And the prevalence of EBV was highest in Asia (35.25%) and lowest in the USA (18.27%) (Huo et al., 2012).

Therefore in this article, we study the prevalence of EBV infection in female breast cancer patients from Modarres Hospital in Iran. 

## Materials and Methods

*Tumor specimens*

This study includes 59 tumor Formalin-Fixed Paraffin-embedded (FFPE) samples selected from the clinical archives of Modarres Hospital, Tehran, Iran. Specimens collected from women diagnosed with BC from 2008 to 2019. Also, we collected 11 non-tumoral specimens. For further studies, we transferred specimens to the school of medicine at Shahid Beheshti University.

*DNA Extraction*

For standard polymerase chain reaction (PCR), genomic DNA (gDNA) was extracted from formalin-fixed paraffin-embedded (FFPE) breast tissues using chemical agents such as xylene. At first FFPE tissues were cut about 10 μm thick using microtome‏ and for removing paraffin from tissue section, we added 1 ml xylene and they mixed on the rotator. Then centrifuged for 5 min and remove the supernatant. Afterward, we repeated these steps one more time. Next, we added 1 ml of 96% ethanol. For drying the ethanol; we put each microtube in heating block 50ºC until ethanol was completely evaporated. Next, we used the digestion buffer and proteinase K solution in each tube. Then we incubated them one overnight.

The next day we put microtubes in 95ºC heater. Then we added phenol and centrifuged them for 5 min and have added the phenol-chloroform solution to each microtube. In the next step, we added only chloroform. In the end, we added ethanol and put them into the incubator for one overnight.

On the third day of extraction, we centrifuged samples for 30 min at maximum speed at 4ºC. Then we discarded SN and left the ethanol to dry in 37ºC heater. At the last step, we added distilled water to them, and then the gDNA extracts were quantified with a Nano Drop spectrophotometer.

*PCR*

The quality control of the extracted DNA was done by using polymerase chain reaction (PCR) for Glyceraldehyde-3-phosphate dehydrogenase (GAPDH) using the specific primers listed in Table2. 

Thirty cycles of GAPDH amplification were performed in a DNA thermal cycler (Bio Intellectica) with the following conditions: denaturing at 95°C for 30 s, hybridization at 55°C for 30 s, and elongation at 72°C for 30s, followed by a final elongation at 72°C for 10 min.

And PCR for EBV was carried out in a total volume of 25 μl using Taq DNA polymerase (Takapouzist, Iran) with the following conditions: 95°C for 5 min; followed by 35 cycles of 95°C, 30 s; 60°C, 30 s; 72°C, 30s; and a final extension at 72 °C for 10 min. Ten microliters of each PCR product was analyzed by electrophoresis in agarose gel. The PCR product size is 250 bp. Reactions containing approximately 25 μl without any DNA were run as negative controls and DNA from B95-8 cell lines as a positive control.

## Results

A total of 70 FFPE samples were included in this study. 59 patients were classified as cases (malignant breast disease), and the remaining 11 as controls. All of the breast cancer samples gave the expected band after amplification with the GAPDH. Then PCR for EBNA-1 was done. Of the 59 cases, 4 were positive for the presence of EBV (6.7%), and No positivity was noted in the control samples. 

The details for the clinical and pathological findings for FFPE breast cancer samples include age group, grading of breast cancer, type of BC, and level of education are collected in Table 1. 

The median age of the patients was about 50 years (range, 29 to 81 years). Our study included cases of female breast cancer only. Histologically, tumors type were lobular (about 5 %) and ductal carcinoma (about 95 %). All positive samples for EBV were ductal type.

We had 4 patients (6.7%) with a low-grade malignancy (tumor grade I), and all remaining had high tumor grades (II, and III) (93.3%). And all positive cases had high grades (II, and III). 57.6% of patients had low educational degrees and even 18.6 % had no degrees and others (23.7%) had high degrees.

**Figure 1:**
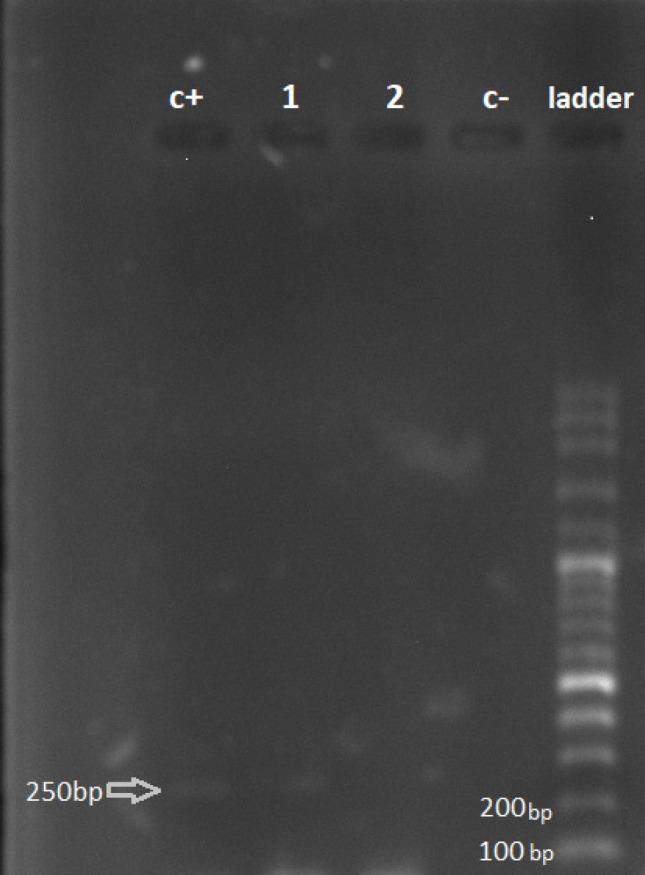
Agarose Gel Electrophoresis of PCR Amplification for the EBNA Gene in BC Patients. Lane 1 shows amplification of 250 bp EBNA gene fragment of EBV from BC patient's sample. Lane NC (negative control), includes distilled water. Lane PC (positive control), includes the B95-8 cell line. Lane L represents a 100bp ladder

**Table 1:** Clinical and Pathological Features of Paraffin-Embedded Samples of Breast Cancer (BC).

Clinical and pathological characteristics of BC patients	Number of patients; N (%)
Age	
Young (<30)	4 (6.7)
Adult (30-50)	19 (32.2)
Old (>50)	36 (61.01)
Grade	
I (low grade)	4 (6.7)
II/ III (high grade)	55 (93.2)
Type of BC	
Invasive lobular	3 (5.08)
Invasive ductal	56 (94.9)
Education degree	
High level	14 (23.7)
Low level	34 (57.6)
No degree	11 (18.6)

**Table 2:** List of Primers Used for the Amplification of GAPDH and EBV Gene Sequences

Gene to be amplified	Primer code	Sequence (5’-3’)
GAPDH (200 bp)	GAPDH-F	ATGTTCGTCATGGGTGTGAA
	GAPDH-R	GGTGCTAAGCAGTTGGTGGT
EBV- EBNA (280 bp)	EBNA-F	TGAATACCACCAAGAAGGTG
	EBNA-R	AGTTCCTTCGTCGGTAGTC

## Discussion

Breast cancer is a multifactorial disease; the role of the infectious agent in this disease is remarkable. The high incidence of breast cancer around the world has got the scientist’s attention to the viral etiology of BC. Though many studies have been done, no etiologic factors for human breast cancer have been known. Recent researches have shown the association of breast cancer and viral infections, such as Epstein–Barr virus (EBV), Human papillomavirus (HPV) and mouse mammary tumor virus (MMTV) (Naushad et al., 2017). The first study of the detection of EBV in breast carcinoma has been described in 1995 by Labrecque LG and his colleagues. They reported 19 positive samples (21%) from 91 cases of breast carcinoma and blood samples by using PCR (Labrecque et al., 1995).

In this study, we considered EBV in BC cases. In the current study, we identified 4 EBV-positive breast tumor cells using PCR for EBNA-1. Our findings are consistent with the results published by others such as the study of Reza MA et al. which was done in the southeast of Iran (Kerman). They revealed the role of EBV in breast carcinoma in 2015. They demonstrated it by using real-time PCR and Immunohistochemistry (IHC) techniques. The presence of EBV was 8/100 (8%), and they stated p53 was suppressed in EBV positive samples (Reza et al., 2015). 

Also, Naushad et al in 2017 could detect EBV in Pakistani breast cancer patients with a prevalence of 24% by using PCR (Naushad et al., 2017). Sharifpour et al., (2019) have observed 27.02% EBV DNA type 1 in the FFPE tissue of Iranian patients with ductal breast carcinoma in 2019 and the detection of EBV DNA was done by nested PCR. 

Ballard in (2015) has shown an EBV presence (39.4%) in ductal and lobular tumor types by EBNA1 staining.

Mario et al., (2010) studied the characterization of EBV Latency Pattern in Argentine Breast Carcinoma. They used IHC with monoclonal antibodies and in situ hybridization to find out EBV genomic DNA and EBNA1 expression in 31% (22/71) of patients while all breast control samples were negative for both viral DNA and EBNA1 protein. And LMP2A was detected in 73% of EBNA1 positive cases (Lorenzetti et al., 2010).

A tissue microarray study was performed in 2015 by Aboulkassim et al., (2015) they could report 51.58% presence of EBV in 108 breast cancer tissues collected from Syrian women. 

Findings of H Arbach et al showed that EBV genomes can be detected by Q-PCR in about half of tumor specimens, usually in low copy numbers. On the other hand, they also found that the viral load is highly variable from tumor to the tumor (Arbach et al., 2006).

Although negative results have also been reported as the study of Glaser et al, which reported negative results in 107 cases using EBER1 transcripts by in situ hybridization, This study included specimens from 21 hospitals in 7 counties from the San Francisco Bay Area of northern California, and it embraced age, sex, and ethnic groups associated with variation in breast cancer incidence in the United States (Glaser et al., 1998).

Similarly, Gaffey et al., (1993) found no evidence of EBV in 16 medullary carcinomas or 18 infiltrating breast carcinomas using PCR.

Also in another study, Lespagnard et al., (1995), couldn’t find EBV in 10 medullary carcinomas by using PCR EBER in situ hybridization, and IHC for LMP1.

Besides in Iran, Dowran et al., (2019) studied on 300 breast biopsy tissues and PCR assay was performed, but they didn’t report any genomic DNA fragment of EBV. 

There is controversy regarding the role of EBV in the pathogenesis of BC. The controversy is influenced by two reasons: technical limitations and duration of fixation. This study demonstrated the presence of EBV genome malignant tumor tissues in women with breast lesions by using PCR assay; more studies need to be analyzed in order to establish the exact role of this virus in the pathogenesis of breast cancer, and utilizing more techniques like Immunohistochemistry or real-time PCR in addition of PCR (Arbach et al., 2006; Joshi et al., 2009; Lorenzetti et al., 2010).

Furthermore, Greer et al., (1991) reported that detection of EBV by PCR in formalin preserved specimens are affected greatly by the duration of fixation. Likewise in our study, we found all DNA positive samples from new specimens collected during 2 years (2018 to 2019). And we couldn’t find any positivity from old specimens (2008 to 2018).

About the grade of malignancy, we had 4 patients with a low-grade malignancy (tumor grade I), and all remaining had high tumor grades (II, and III). And all positive specimens for the EBV had a high-grade tumor. One of them was grade II and three of them were grade III. So we can conclude from our results and other articles, there is an association between the presence of EBV and the grade of tumors (Bonnet et al., 1999; Ribeiro-Silva et al., 2004; Preciado et al., 2005).

Income and education have a strong association with the incidence of breast cancer (Devesa and Diamond, 1980), for example, Liu et al., (2017) performed a multi-center 10-year epidemiological study which determined the impact of the education level of Chinese female breast cancer (4,211 cases). For patients within the lower educational group, the tumor grade was higher and the rates of investigations were lower, as were the rates of radiotherapy, chemotherapy, and endocrine therapy. Similarly, in our study, most of the patients had a low level of education. So there is a very urgent need for regular learning courses for the practice of breast cancer screening especially for that person with less educational level.

In conclusion, according to our findings and review of other studies, it can be determined that EBV may have an etiologic role in breast cancer. In our study, all positive samples were in a high grade of the tumor. So we can conclude, there is a relationship between the presence of EBV and the grade of the tumor. Higher grades show more possibility of the presence of EBV. Another factor was the level of education, which has an important role in the check-up and follows up during the disease. Also, all of the positive cases were women with a low level of education.

Thus our results with 6.7% EBV-DNA positivity supports the possibility of an etiologic role of EBV in the induction and development of BC, and other factors such as age, level of education, the grade of tumor and new techniques should be considered. So more studies using more specific and sensitive techniques are needed.
